# What Regional Living Conditions Affect Individual Smoking of Adults in Russia

**DOI:** 10.3389/ijph.2021.599570

**Published:** 2021-04-01

**Authors:** Sergey A. Maksimov, Svetlana A. Shalnova, Yulia A. Balanova, Vladimir A. Kutsenko, Svetlana E. Evstifeeva, Asiia E. Imaeva, Oksana M. Drapkina

**Affiliations:** Department of Epidemiology of Chronic Non-Communicable Diseases, National Medical Research Center for Therapy and Preventive Medicine of the Ministry of Healthcare of the Russian Federation, Moscow, Russia

**Keywords:** smoking, regional environment, health geographic, multi-level study, Russia

## Abstract

**Objectives:** Our study evaluated the impact of a wide range of characteristics of large administrative regions on the individual level of cigarette smoking in the Russian adult population.

**Methods:** The pool of participants included 20,303 individuals aged 25–64 years. We applied 64 characteristics of the 12 Russian regions under study for 2010–2014. Using principal component analysis, we deduced five evidence-based composite indices of the regions. We applied the generalized estimating equation to determine associations between the regional indices and the individual level of smoking.

**Results:** The increased Industrial index in the region is associated with the probability of smoking (odds ratio = 1.15; 95% confidence interval = 1.06–1.24). The other indices show associations with smoking only in separate gender and educational groups. Surprisingly, it was found that the Economic index has no associations with the probability of smoking.

**Conclusion:** We evaluated the key associations of the territorial indices with the individual probability of smoking, as well as the mutual influence between the territorial indices and individual factors.

## Introduction

Smoking causes the death of millions of people every year. Specifically, in 2017, about eight million people worldwide died of tobacco-related diseases (1). Numerous international prospective studies and meta-analyses attest to the significant role of smoking in the development of a wide range of serious chronic non-communicable diseases: Coronary heart disease and brain hemorrhage (2), diabetes (3), and oncological diseases (4). According to the World Health Organization, the global trend consists of a decreasing degree of smoking incidence throughout the world: In 2000, 33.3% of the world population was smoking; by 2015, however, this figure dropped to 24.9%. At the same time, the dynamic of such a decrease varies significantly depending on demographic and regional characteristics (5). The degree of smoking incidence among men in the majority of high-income countries began to decline in the mid-1990s, while the degree of smoking incidence in many low and middle-income countries remained unchanged or even increased. The degree of smoking incidence among women has also decreased in most countries, although the decline began later and was slower compared to that of men (6).

At the individual level, smoking addiction significantly varies depending on gender, age, and a number of socio-economic characteristics, such as education, income level, marital status, and profession (5, 7, 8). However, even with adjustments for individual characteristics, there are territorial differences in the degree of smoking incidence depending on the scale: International (7, 8), regional (9), administrative, and district (10).

In the mid-1990s, several leading epidemiologists started emphasizing the need to switch from the phenomenology of the prevalence of non-communicable diseases and their risk factors, to the development of procedures for identifying associated cause-and-effect relationships (11–13). This gave a boost to the study of new epidemiological theories, including the socio-ecological model, which provides for biological and social production of diseases as equally significant components. This resulted in a large number of epidemiological surveys that studied the multi-level impact of territorial characteristics on individual health. For the past two decades, there have also been numerous studies of how the territory of residence affects the smoking status irrespective of individual characteristics (14, 15). It is significant that the impact of territorial living conditions on individual health indicators can vary considerably depending on the scale of the selected territory (16–19). Most of the “smoking geography” studies focus on small territorial entities: Postal index zones, city districts, municipalities, and areas with a population of under 50,000–100,000 people (14, 15, 20–27). There are considerably fewer works devoted to the research of territorial smoking predictors at the international level (28–30). Moreover, studies of “smoking geography” covering territorial entities between countries and small areas, that is, large regions, provinces, and states within a country, are even more sporadic (31–33). Apparently, such studies are likely to be of interest mostly for countries with a large territory.

Such surveys of “smoking geography” essentially focus on studying one or several social or economic characteristics selected as territorial predictors: The Gini index (23, 28, 31, 33), crime rate (20), Gross Domestic Product (28, 30), education level (21), per capita income (22, 23), unemployment rate (22), and poverty headcount (34). However, in reality, there are numerous complex relationships between territorial characteristics (17, 35). Therefore, associations of a specific territorial characteristic with health indicators may not indicate a cause-and-effect relationship, but a relationship mediated by other territorial characteristics. Thus, many researchers of “smoking geography” apply composite socio-economic indices calculated based on several social and economic territorial characteristics at once (14, 15, 24, 25, 28, 32). These studies allow to evaluate socio-economic territorial predictors of health status. However, despite the fact that the socio-economic environment is viewed as one of the most significant predictors of health status (and smoking in particular), and rightfully so, one, nevertheless, cannot but take into account the possible impact of other territorial characteristics. Once this line of reasoning is applied, it may be of interest to focus on those few studies that have employed an empirical approach to evaluate territorial characteristics, with subsequent analysis of their impact on individual health indicators (36), including smoking status (26, 27).

Finally, as part of the rationale for this study, it should not go unspoken that in Russia, there has been no analysis of the impact of territorial characteristics on individual health indicators, including smoking. A certain number of studies show geographical differences in the degree of smoking incidence (37, 38); moreover, they show a geographical trend of the degree of smoking incidence in Russia increasing from south to north and from west to east (39, 40). However, the reasons for such patterns have not been analyzed.

Thus, although it has been found that the socio-economic environment affects smoking habits, there is, however, no convincing evidence with regard to large territorial entities and other, non-socio-economic, characteristics. With this study, we aimed to evaluate the impact of the characteristics of large administrative regions on individual probability of smoking in the Russian adult population. Herein, we intended to find answers to the following questions: 1) What main groups (indices) of characteristics that describe Russian regions from different perspectives—geographic, demographic, social, economic, industrial, environmental, etc.—can be singled out? 2) What and how do regional living conditions affect the individual probability of smoking in the Russian population? 3) Do regional living conditions have any particular impact on smoking status depending on individual characteristics of the Russian people; in other words, are there any interactions between regional and individual levels?

## Methods

### Sample Description

For the purpose of analysis, we used data from the cross-sectional phase of the epidemiological study “Epidemiology of Cardiovascular Diseases in the Regions of the Russian Federation” (ESSE-RF) conducted in 2013–2014. A total of 21,923 individuals aged 25–64 years were examined. More detailed information on the ESSE-RF sampling and study protocol was given previously (41). In brief, the study was conducted in 13 regions of the Russian Federation. The sample was drawn based on the Kish method, which provides for systematic, multi-step, random community-based sampling on the premises of medical and preventive treatment facilities. The study was carried out in accordance with the standards of Good Clinical Practice and the principles of the Declaration of Helsinki. The study protocols were approved by the Ethics Committee of the National Medical Research Center for Therapy and Preventive Medicine (Moscow), the National Medical Research Center of Cardiology (Moscow), and the Almazov National Medical Research Centre (St. Petersburg), as well as by collaborating centers in the regions where this study was conducted. All participants gave their written informed consent prior to being included in the study. The response rate was approximately 80%, with some variations across the study regions.

**FIGURE 1:**
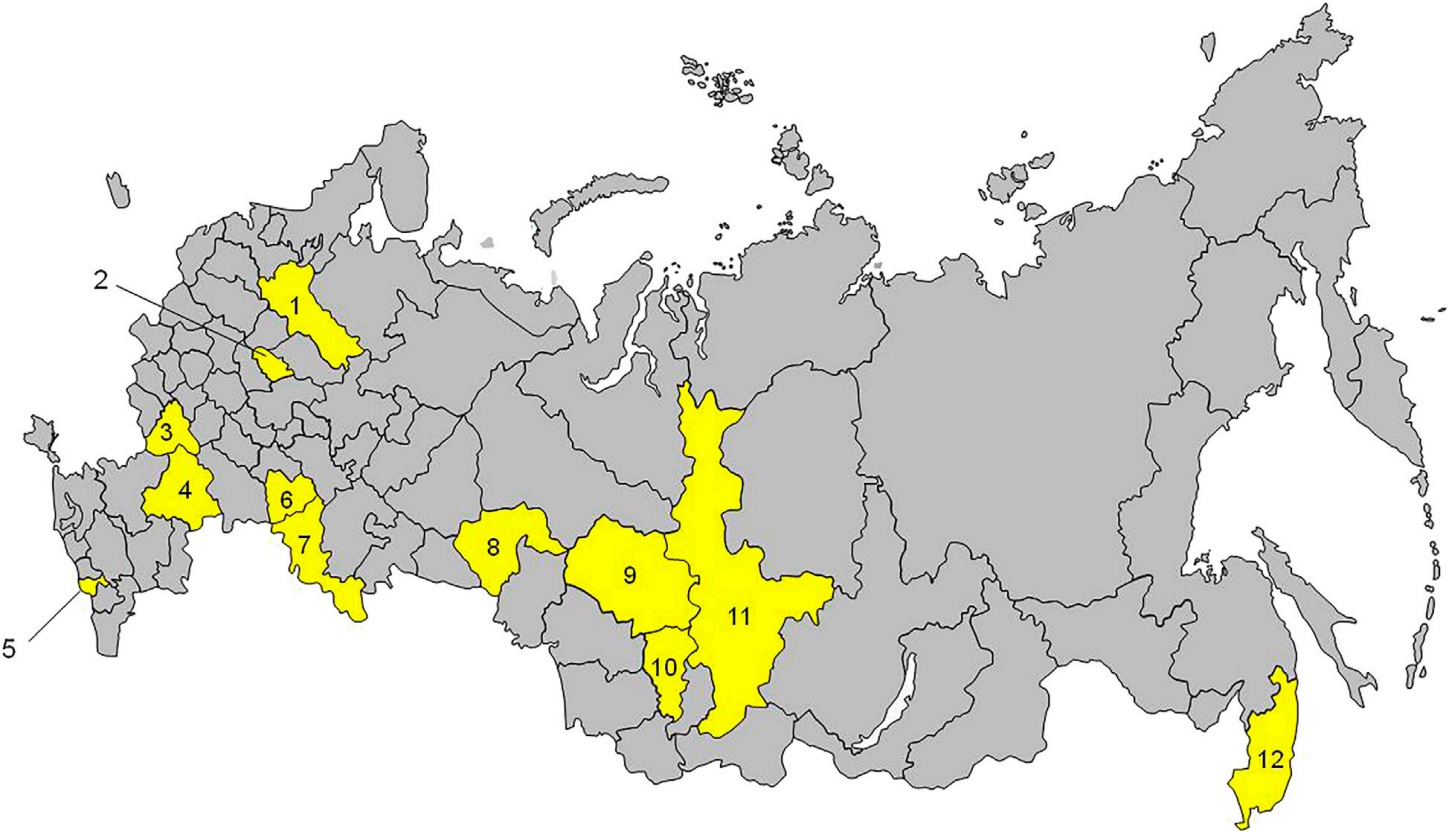
Regions of Russia participating in the study (“Epidemiology of Cardiovascular Diseases in the Regions of the Russian Federation”, Russia, 2013–2014). 1, Vologda; 2, Ivanovo; 3, Voronezh; 4, Volgograd; 5, Vladikavkaz; 6, Samara; 7, Orenburg; 8, Tyumen; 9, Tomsk; 10, Kemerovo; 11, Krasnoyarsk; 12, Vladivostok.

St. Petersburg (1,588 people) was excluded from the final sample, since its regional characteristics are substantially different from those of the other 12 regions. The city of St. Petersburg is classified as a separate administrative territory in the Russian Federation, while the other 12 regions are large territories that include both urban and rural areas. Figure 1 shows the geographical location of the 12 regions participating in the study.

Another 32 individuals (0.2%) without a recorded smoking status were excluded from the remaining sample. As regards other individual factors, 265 people (1.3%) did not provide data on income, 152 individuals (0.7%) on marital status, and 15 individuals (0.1%) on the level of education. The missing data for these three factors were reconstructed using the k-nearest neighbor algorithm. Data imputation was carried out based on the input parameters, i.e., region, location, gender, and age. Thus, the final sample size with complete data (i.e., initial and reconstructed) amounted to 20,303 individuals. The general characteristics of the sample are given in Table 1.

**TABLE 1:** Baseline characteristics of the study participants (“Epidemiology of Cardiovascular Diseases in the Regions of the Russian Federation”, Russia, 2013–2014).

Characteristics		*n*	Smoking		*p*-value
			*n*	%	
Summary		20,303	4,427	21.8	–
Gender	Women	12,511	1,407	11.2	<0.001
	Men	7,792	3,020	38.8	
Age	25–34 years	4,259	1,252	29.4	<0.001
	35 44 years	4,054	1,058	26.1	
	45–54 years	5,660	1,206	21.3	
	55–64 years	6,330	911	14.4	
Location	Urban	16,123	3,503	21.7	0.60
	Rural	4,180	924	22.1	
Income	Low	3,432	693	20.2	<0.001
	Middle	13,438	2,892	21.5	
	High	3,433	842	24.5	
Education	Non-university	11,726	2,925	24.9	<0.001
	University	8,577	1,502	17.5	
Family	No	7,093	1,396	19.7	<0.001
	Yes	13,210	3,031	22.9	
Region	Krasnoyarsk	1,521	387	25.4	<0.001
	Vladivostok	2,107	459	21.8	
	Volgograd	1,457	298	20.4	
	Vologda	1,617	394	24.4	
	Voronezh	1,585	352	22.2	
	Ivanovo	1872	346	18.5	
	Kemerovo	1,611	490	30.4	
	Samara	1,587	301	19.0	
	Orenburg	1,578	366	23.2	
	Tomsk	1,593	450	28.2	
	Tyumen	1,639	240	14.6	
	Vladikavkaz	2,136	344	16.1	

### Individual Variables and Responses

From individual variables, we selected the socio-economic and demographic characteristics as having the highest evidenced impact on smoking. These included gender, age, level of education (at non-university/university level), marital status (family/no family), and income level. Location was represented by urban or rural area. The income level was evaluated indirectly based on three questions characterizing the share of income spent on food, the respondents’ opinion on the family budget, and prosperity as compared to other families. Each question offered five possible answers that ranged in points from 1 (the “poorest” answer) to 5 (the “richest” answer). The point total was used to calculate terciles, which in turn were used to break the income level down into three categories: “Low,” from 3.0 to 7.2 points; “Middle,” from 8.0 to 10.3 points; and “High,” from 11.0 to 15.0 points. The current smoking status was construed as a response: Smokes (smoking of one or more cigarettes per day) or does not smoke (including quitted smoking). All individual variables were obtained by means of face-to-face interviews.

### Regional Variables

We used figures provided on the official website of the Federal State Statistics Service of Russia for the 12 regions of the Russian Federation under study that give a measure of the following aspects: Climatic and geographic (4 factors), demographic (10 factors), social (13 factors), economic (9 factors), environmental (2 factors), industrial (6 factors), medical and infrastructure (3 factors), health status of the population (10 factors), and alcohol consumption (6 factors). The majority of the figures provided were for the period 2010–2014, except for gross regional product and per capita household consumption, which were for the period 2010–2013. Regional characteristics were averaged over the given period; a total of 64 regional characteristics were analyzed. A complete definition, average, minimum and maximum values, and standard deviations of all of the regional characteristics used are presented in Supplementary Table S1.

Regional characteristics are substantially interconnected, both contextually and statistically. In order to reduce the dimensionality of the data, we used principal component analysis (PCA), which is widely used to determine the composite indices of territories of residences based on several reference characteristics (14, 35, 42–44). Our aim was to derive a parsimonious set of factors that captures the shared variance of a broad spectrum of region structural characteristics. We used varimax orthogonal rotation to simplify the structure of factors and to improve their interpretability. We identified factors with a share of explained variance of over 5%, with subsequent assessment of the gap according to the Cattell scree plot. Since there is a strong correlation between regional characteristics, we recognized a factor loading of | ≥0.65 | as a substantial contribution to the structure of factors. All of the resulting factor scores were standardized to have a mean of zero and a standard deviation of one.

We identified a total of five integrated factors (Table 2) that explain a cumulative 77.6% of the total variance. The first factor comprises ten characteristics: Average per capita consumption of vodka, wine, low-alcohol beverages and brandy, average annual air temperature (negative load on the factor), timberland area, per capita crime rate, geographical latitude of the regional center, proportion of decrepit and dilapidated housing, and proportion of students in the second and third shifts. The second factor comprises five characteristics: Natural population growth (negative load), birth (negative load) and mortality rates, proportion of population over unemployable age, and mortality from respiratory diseases. The third factor comprises eight characteristics: Rates of production of minerals and electric power, mortality from tuberculosis, infectious diseases and external causes, proportion of people in the region working under harmful working conditions, population size of the region, and emissions into the atmosphere. The fourth factor comprises five characteristics: Number of people employed in fisheries, average per capita volume of paid services, average per capita number of cars, male/female ratio (negative load), and geographical longitude of the regional center. The fifth factor comprises five characteristics: Per capita retail turnover, per capita household consumption, Gini index, per capita income of the population, and level of manufacturing in the region. These factors are easily interpreted, except for factor 4. Given the prevailing loads and for the purposes of this study, we named the identified factors as follows: Factor 1—Socio-geographic index; factor 2—Demographic index; factor 3—Industrial index; factor 4—Mixed index; and factor 5—Economic index.

**TABLE 2:** Factor loadings of the principal regional indices identified (“Epidemiology of Cardiovascular Diseases in the Regions of the Russian Federation”, Russia, 2013–2014).

Characteristic	Factors (Indices)				
	1	2	3	4	5
% from total variance	28.0	16.9	12.5	10.4	9.8
Sales of vodka	0.95				
Average annual temperature	−0.88				
Timberland area	0.82				
Sales of wine-making products	0.80				
Number of recorded crimes	0.76				
Location of the regional center, north latitude	0.71				
Decrepit and dilapidated housing	0.69				
Portion of students second and third shifts	0.69				
Sales of low-alcohol beverages	0.67				
Sales of brandy and brandy spirits	0.66				
Natural increase rate		−0.99			
Crude birth rate		−0.94			
Population of unemployable age		0.92			
Crude mortality rate		0.91			
Mortality rate from diseases of the respiratory system		0.70			
Mineral extraction			0.90		
Mortality rate from tuberculosis			0.80		
Electric power production			0.79		
Mortality rate from infections			0.79		
Portion of people employed at toxic and (or) hazardous jobs			0.78		
Mortality rate from external causes			0.73		
Population size			0.71		
Emissions of pollutants into the atmosphere			0.67		
Number of employees of fisheries				0.95	
Per capita amount of paid services				0.95	
Number of private passenger cars				0.78	
Male/female ratio				−0.77	
Location of the regional center, east longitude				0.69	
Per capita retail turnover					0.92
Per capita actual final consumption of households					0.91
Gini Index					0.88
Per capita income per month					0.84
Manufacturing					0.76

### Statistical Analysis

We used bivariate statistics (proportions and chi-squared test) to summarize the characteristics of the samples and the distribution of the socio-economic status indicators. We used Pearson correlation to assess the interconnections between regional characteristics. The survey data were represented by a complex two-level sample with individual and regional characteristics, which requires the application of appropriate methods of statistical analysis. Studies based on complex cluster samples commonly use mixed regression models (for example, a generalized linear mixed model); however, a number of surveys have proven that application of marginal approaches that provide for a more robust and valid inference can also be successful (45, 46). Therefore, we used the generalized estimating equation (GEE) with constant standard errors to determine associations between regional indices and individual smoking levels, with due regard to the nested data structure (i.e., individuals in the regions). We completed several sets of logistic models of smoking probability that included a calculation of the odds ratio (OR) and the Wald statistic. The “zero” model included individual variables only. Model 1 included individual variables and all regional indices. Next, we evaluated interactions of the most important individual variables (i.e., gender, age, and level of education) and all of the regional indices. Since we detected substantial interactions between the individual variables of “gender” and “level of education” and the regional indices, we performed a separate analysis of Model 1 for men/women and various levels of education for the purpose of a better interpretation of such interactions. For the purposes of descriptive statistics, two-way analysis of categorical variables, correlation analysis, and PCA, we used Statistica Version 10.0 (Statsoft Inc., United States), and for the GEE, we used SPSS Version 22 (IBM Corp., United States).

## Results

The “zero” model showed a impact of gender, level of education, and age on the probability of smoking (Table 3). Model 1, which also includes the regional indices, showed a impact of individual income level. With regard to the regional indices, an increased probability of smoking is associated with an increased Socio-Geographic index (OR = 1.12: 1.03–1.22) and an increased Industrial index (OR = 1.15: 1.06–1.24). Chi-squared estimates of the likelihood Type III test are as follows, in descending order: age, 143.9; level of education, 104.2; gender, 90.1; Industrial index, 12.9; income level, 8.6; and Socio-geographic index, 7.0.

**TABLE 3:** Multivariate association of individual and regional characteristics with smoking (“Epidemiology of Cardiovascular Diseases in the Regions of the Russian Federation”, Russia, 2013–2014).

Characteristic		Model 0			Model 1		
		OR	95% CI	*p*-value	OR	95% CI	*p*-value
Gender (ref. Women)	Men	4.95	3.54–6.91	<0.001	4.91	3.53–6.81	<0.001
Location (ref. Urban)	Rural	1.00	0.80–1.25	0.98	1.02	0.81–1.29	0.86
Income (ref. Low)	Middle	0.87	0.74–1.02	0.081	0.84	0.73–0.96	0.013
	High	0.84	0.69–1.02	0.085	0.78	0.65–0.92	0.004
Education (ref. Non-university)	University	0.53	0.47–0.59	<0.001	0.53	0.47–0.60	<0.001
Family (ref. No)	Yes	0.94	0.88–1.01	0.10	0.95	0.89–1.01	0.094
Age		0.97	0.96–0.98	<0.001	0.97	0.96–0.98	<0.001
Socio-geographical index					1.12	1.03–1.22	0.008
Demographic index					1.00	0.88–1.14	0.99
Industrial index					1.15	1.06–1.24	<0.001
Mixed index					1.00	0.96–1.05	0.92
Economic index					0.93	0.83–1.04	0.19

We detected interactions between the regional indices and individual factors in terms of their impact on the probability of smoking (Table 4). There is interactions between gender and all of the regional indices, except for the Economic index, between age and the Socio-geographic index, and between education and the Socio-geographic and Mixed indices. Judging by the OR values, the impact of the indices on individual smoking is weaker or negative for men, people with university-level education, and people of older age.

**TABLE 4:** Interaction effects on smoking individual and regional characteristics (“Epidemiology of Cardiovascular Diseases in the Regions of the Russian Federation”, Russia, 2013–2014).

Interaction	Parameters of Models		
	OR	95% CI	*p*-value
Gender			
SGI^a^ Men (ref. SGI^a^ Women)	0.65	0.60–0.70	<0.001
DemI^a^ Men (ref. DemI^a^ Women)	0.77	0.71–0.84	<0.001
IndI^a^ Men (ref. IndI^a^ Women)	0.87	0.79–0.97	0.013
MixI^a^ Men (ref. MixI^a^ Women)	0.78	0.75–0.80	<0.001
EcoI^a^ Men (ref. EcoI^a^ Women)	0.96	0.85–1.07	0.43
Age			
SGI^a^ Age	1.005	0.999–1.010	0.039
DemI^a^ Age	0.999	0.996–1.002	0.55
IndI^a^ Age	0.999	0.994–1.003	0.63
MixI^a^ Age	0.999	0.998–1.001	0.48
EcoI^a^ Age	0.996	0.992–1.001	0.11
Education			
SGI^a^ University (ref. SGI^a^ Non-university)	0.86	0.76–0.98	0.027
DemI^a^ University (ref. DemI^a^ Non-university)	0.99	0.90–1.10	0.92
IndI^a^ University (ref. IndI^a^ Non-university)	0.99	0.89–1.11	0.88
MixI^a^ University (ref. MixI^a^ Non-university)	0.92	0.85–0.99	0.023
EcoI^a^ University (ref. EcoI^a^ Non-university)	1.06	0.97–1.15	0.20

All Models includes individual variables and all regional indices.

^a^ SGI, Socio-geographical index; DemI, Demographic index; IndI, Industrial index; MixI, Mixed index; EcoI, Economic index.

Separate modeling shows that gender is a substantial factor in terms of the associations between individual smoking probability and characteristics of the region of residence (Table 5). Thus, the direct impact of the Socio-geographic and Demographic indices is typical for women only (respectively, OR = 1.47: 1.35–1.60 and OR = 1.18: 1.01–1.36), but not for men. The Industrial index directly affects the probability of smoking in both gender groups; however, the impact is more substantial for women (OR = 1.21: 1.10–1.33) than for men (OR = 1.08: 1.01–1.16). The Mixed index shows opposite trends based on gender: A direct impact on women (OR = 1.16: 1.10–1.22) and the reverse on men (OR = 0.90: 0.86–0.94).

**TABLE 5:** Multivariate association of individual and regional characteristics with smoking (gender stratification) (“Epidemiology of Cardiovascular Diseases in the Regions of the Russian Federation”, Russia, 2013–2014).

Characteristic		Women			Men		
		OR	95% CI	*p*-value	OR	95% CI	*p*-value
Location (ref. Urban)	Rural	0.97	0.77–1.21	0.76	1.06	0.81–1.39	0.69
Income (ref. Low)	Middle	0.77	0.67–0.87	<0.001	0.84	0.70–1.01	0.056
	High	0.79	0.64–0.97	0.026	0.77	0.64–0.93	0.007
Education (ref. Non-university)	University	0.54	0.45–0.65	<0.001	0.50	0.44–0.56	<0.001
Family (ref. No)	Yes	0.77	0.69–0.86	<0.001	1.07	0.97–1.19	0.18
Age		0.95	0.94–0.96	<0.001	0.98	0.97–0.99	<0.001
Socio-geographical index		1.47	1.35–1.60	<0.001	0.95	0.87–1.03	0.24
Demographic index		1.18	1.01–1.36	0.031	0.91	0.81–1.02	0.12
Industrial index		1.21	1.10–1.33	<0.001	1.08	1.01–1.16	0.027
Mixed index		1.16	1.10–1.22	<0.001	0.90	0.86–0.94	<0.001
Economic index		0.97	0.84–1.12	0.66	0.92	0.82–1.02	0.11

In terms of the level of education (Table 6), the direct impact of the Socio-geographic index is typical for non-university education (OR = 1.19: 1.09–1.29), but not for university-level education. An inverse association between the Mixed index and the probability of smoking, being close to statistically significant, is found in individuals with university-level education (OR = 0.97: 0.94–1.00), but not with non-university education. It should be noted that the associations between the Industrial index and the probability of smoking are equal in their direction and intensity for both gradations of education.

**TABLE 6:** Multivariate association of individual and regional characteristics with smoking (education stratification) (“Epidemiology of Cardiovascular Diseases in the Regions of the Russian Federation”, Russia, 2013–2014).

Characteristic		Non-university level			University level		
		OR	95% CI	*p*-value	OR	95% CI	*p*-value
Location (ref. Urban)	Rural	1.08	0.86–1.37	0.50	0.88	0.67–1.16	0.37
Income (ref. Low)	Middle	0.83	0.72–0.96	0.011	0.80	0.65–0.98	0.032
	High	0.81	0.62–1.05	0.11	0.72	0.57–0.91	0.006
Gender (ref. Women)	Men	5.35	3.96–7.23	<0.001	4.21	2.86–6.19	<0.001
Family (ref. No)	Yes	0.99	0.89–1.09	0.78	0.87	0.76–0.99	0.036
Age		0.96	0.95–0.97	<0.001	0.98	0.97–0.99	<0.001
Socio-geographical index		1.19	1.09–1.29	<0.001	1.04	0.93–1.17	0.49
Demographic index		1.00	0.87–1.15	0.99	1.01	0.89–1.13	0.94
Industrial index		1.15	1.05–1.25	0.002	1.13	1.04–1.24	0.007
Mixed index		1.04	0.97–1.12	0.24	0.97	0.94–1.00	0.051
Economic index		0.91	0.80–1.02	0.10	0.97	0.87–1.09	0.64

## Discussion

The results we obtained allowed us to answer all of the questions posed, and provided an overall measure of the impact of region-specific characteristics on the degree of smoking incidence. Based on the 64 territorial characteristics, we determined five latent factors that form the main part of the variance: The Socio-geographic, Demographic, Industrial, Mixed, and Economic indices. Of these, the Economic index, surprisingly, showed no associations with the probability of smoking, in either the total sample size or the stratification analyses.

The Industrial index shows the most consistent associations, both in terms of the primary effects and in interactions with individual factors. Living in regions with a high level of mineral extraction, electric power production, poor labor conditions for the majority of workers (which may be related to the high mortality rate from external causes), and high levels of emissions into the atmosphere from stationary sources increases the individual probability of smoking.

The Socio-geographic, Demographic, and Mixed indices show substantial interactions with gender (most intense), age, and level of education. There are associations between the high levels of alcohol consumption in the region, accompanied by a high crime rate and deterioration of certain social conditions (i.e., quality of housing and the educational environment for children), as well as the climatic and geographic location of the regions (i.e., further to the north and colder). Living in such regions is associated with an increased individual probability of smoking among women, people with low-level education or qualifications, as well as older people.

Living in demographically depressed regions with a low birth rate, a high mortality rate, and, as a result, a negative rate of natural increase, as well as a large proportion of older people in the general population, is associated with an increased individual probability of smoking among women.

Finally, the Mixed index is the most incomprehensible, both in terms of its interpretation and its correlation with the probability of smoking. We found correlations between the development of fisheries and fish farms, the high volume of paid services in the region, the large number of private cars in the region, as well as the increased proportion of women in the general population, as well as the geographic location of the region (i.e., eastern longitude). Living in such regions increases the probability of smoking among women and, on the contrary, reduces it among men and people with university-level education.

The contribution of the selected territorial indices to the individual probability of smoking is rather small, especially in comparison to the substantial contribution of individual factors (i.e., age, level of education, and gender). This is consistent with the results of other multi-level studies of the degree of smoking incidence (22, 26). Nevertheless, this contribution is statistically significant, and may be of interest for the practical development of preventive measures and for monitoring of the degree of smoking incidence in administrative territories.

### Comparison to Similar Multi-Level Studies

Conducting a comparative analysis of the obtained results and the results of other similar multi-level studies is not straightforward, since there are substantial differences in terms of the analyzed territorial characteristics and/or the indices determined on such a basis. First, it should be noted that there is a rather high level of differentiation for four of the five regional indices that we determined. Thus, it is not only the widely analyzed social and economic distinguishing characteristics, but also other regional characteristics that are not usually studied in such surveys that make a significant contribution to the overall variance. These are geographic characteristics, in terms of the industrial, environmental, and certain demographic factors. Most commonly, multi-level studies are based on either specific socio-economic characteristics (for example, the Gini index or the crime rate) or on a priori calculated composite socio-economic indices (for example, the Swedish Care Need Index (47) or the Japanese Areal Deprivation Index (25). Even those few studies that use an empirical assessment of territorial characteristics include mostly socio-economic characteristics in their fundamental lists (26, 27).

Studies covering districts, boroughs, and municipalities often show no associations between smoking and socio-economic characteristics, for example, the crime rate (20), the unemployment rate (22), the average per capita income (22, 23), or the Gini index (23). Furthermore, a number of studies, surprisingly, show positive associations between socio-economic characteristics and smoking, which means that an improvement of the socio-economic situation caused an increase in the degree of smoking incidence (14, 26). However, a rather large number of studies show the traditionally expected increase in the degree of smoking incidence amid the deteriorating socio-economic environment in terms of specific characteristics (22, 34) and, in particular, in terms of the composite socio-economic indices (15, 25, 27, 47).

At the same time, all of the few studies covering the territorial level of states and provinces show no correlation between the probability of smoking and socio-economic characteristics, for example, the Gini index for adolescents (33) and elderly people (31) and the socio-economic index for pregnant women (24). We also found that the Economic index did not show a correlation with the probability of smoking, either in terms of the total sample size or in terms of the specific characteristics of gender, age, and level of education. At the same time, the contribution of a number of social characteristics is evident, especially for women.

### Review of the Results Obtained in Other Russian Studies

As noted above, earlier multi-center epidemiological studies in Russia (in 1993 and 2003–2004) showed a shift in the degree of smoking incidence toward the northern and eastern regions of the country (39, 40). The subsequent study conducted in 2013–2014 (we used the same individual data for our analysis) showed a slight change in the epidemiological situation in terms of descriptive statistics (38, 39). It showed an increased degree of smoking incidence from north to south and from west to east among men, and from south to north and from west to east among women. Certainly, the geographical location itself cannot be a predictor of the degree of smoking incidence, and the observed trends are also dependent on other regional characteristics. Our results are a “step forward” toward understanding these trends. First, we applied a statistical method that allows for reliable evaluation of complex data with a hierarchical (nested) structure, which increases the reliability of the “smoking geography” evaluation. Second, based on the latent factors identified with the help of PCA, the geographic characteristics correlate with other regional characteristics that presumably determine territorial associations with the degree of smoking incidence. The results show that the increased probability of smoking among women, as well as among older people in the northern regions of Russia, is associated with the respective deterioration of the social environment and, in particular, with the high levels of alcohol consumption, crime rate, and poor quality of housing, as well as the educational environment for children. At the same time, from west to east, the probability of smoking increases among women, but decreases among men and people with university-level education. This trend is also associated with the other territorial characteristics included in the Mixed index, which, unfortunately, is difficult to interpret.

### Advantages and Limitations

This study is the first Russian multi-level analysis of hierarchical data on the “geography” of the degree of smoking incidence. For that matter, this study is the first Russia multi-level analysis of the “geography” of any health indicator whatsoever. We performed this analysis based on a large amount of data. In order to obtain scientific evidence, we used modern suitable methods of statistical analysis, for example, the GEE, for multi-level analysis purposes.

It should be noted that our empirical approach to the identification of territorial indices has not yet been used widely enough in similar studies. From this perspective, our study provides new scientific data, including for the purposes of the possible application of new methodological approaches in this particular scientific field.

Finally, it should be noted that we used an evaluation of the interactions between the impact of the territorial indices and individual factors on the probability of smoking for the purposes of our work. This allowed us to clarify the identified patterns in terms of the total sample size (that is, the key effects). Such evaluation of the interactions between the territorial and individual factors is rarely found in similar studies.

It should be noted that the shortcomings of the analysis include the relatively small number of regions (12 in total), that is, territorial entities under study. Clearly, this weakens the analysis in terms of using poor quantitative scales of territorial characteristics, which in turn may affect the resulting territorial indices, as well as the analysis of the key effects and the interactions between the territorial indices and the probability of smoking. On the other hand, such surveys are not unusual, especially when studying large territorial entities (33).

Another shortcoming is that the analysis also resulted in the applicability of a Mixed index that is difficult to interpret. However, obtaining such latent factors that are difficult to interpret is often the “flip side of the coin” of using statistical methods to reduce the dimensionality of data in the context of empirical approaches. It should be noted that the “socio-economic indices,” the “deprivation indices,” and the “district welfare indices” widely applied in similar studies also often use PCA to obtain the first key factor speculatively designated as socio-economic.

### Summary

The analysis results allowed us to evaluate the impact of regional characteristics on the individual probability of smoking in a cross-sectional study of the Russian population. Based on the data collected by the official state statistics authorities of the Russian Federation, we determined the main groups (indices) of characteristics describing the Russian regions from different perspectives. The empirical approach we applied to determine the territorial indices is still quite new and poorly known in the context of multi-level studies, which means that this study provides new scientific data. We evaluated the key associations of the territorial indices with the probability of individual smoking, as well as interactions between the territorial indices and individual factors (i.e., gender, age, and level of education). The results we obtained provide, for a first-time, multi-level evaluation of the health status in Russia from the perspective of environmental epidemiology. Furthermore, this allowed us to provide a well-founded description of the “smoking geography” in Russia and to add the Russian results to the pool of similar global data. Since the degree and the dynamic of smoking incidence in Russia make this country one of the most adverse in the world (along with other countries of Eastern Europe), the results we obtained will be of interest for the purposes of healthcare management and preventive medicine in Russia.

## Data Availability

The datasets presented in this article are not readily available because of prohibition of transferring data to third parties. Requests to access the datasets should be directed to Svetlana Shalnova, sshalnova@gnicpm.ru.
