# Supplementary material for: What Regional Living Conditions Affect Individual Smoking of Adults in Russia
Source: Int J Public Health. 2021 Apr 1;66:599570. doi: 10.3389/ijph.2021.599570 (PMC8565254; doi:10.3389/ijph.2021.599570)
Supplement: Supplementary file 1 [file Table1.DOCX]

Supplementary Material 1. Baseline characteristics of the regional characteristics (“Epidemiology of Cardiovascular Diseases in the Regions of the Russian Federation”, Russia, 2013-2014).

| Characteristics | Mean | Standard Deviation | Minimum | Maximum |
| --- | --- | --- | --- | --- |
| **Geography and Climate** | | | | |
| Average annual temperature, degrees Celsius | 4.6 | 3.1 | 0.9 | 10.4 |
| Average annual amount of precipitation, millimeters of mercury | 568 | 166 | 347 | 929 |
| Location of the regional center, north latitude, degrees | 52 | 5 | 43 | 59 |
| Location of the regional center, east longitude, degrees | 64 | 28 | 39 | 131 |
| **Demographic Landscape** | | | | |
| Population size at the year-end*, thousand people | 1923.0 | 831.0 | 707.4 | 3213.2 |
| Urban population at the year-end*, % | 72.6 | 8.1 | 59.8 | 85.5 |
| Male/female ratio at the year-end*, women per 1000 men | 1160 | 37 | 1085 | 1232 |
| Population of unemployable age at the year-end*, % | 23.1 | 2.4 | 19.7 | 26.9 |
| Demographic burden*, number of people of unemployable age per 1000 people of employable age | 671 | 36 | 612 | 716 |
| Natural increase rate*, per 1000 people | -0.85 | 3.30 | -6.14 | 4.40 |
| Net migration rate*, per 10,000 people | 4.9 | 43.5 | -60.8 | 94.4 |
| Marriage/divorce ratio*, number of divorces per 1000 marriages | 547 | 60 | 392 | 614 |
| Crude birth rate*, number of newborns per 1000 people | 13.1 | 1.8 | 10.6 | 16.5 |
| Crude mortality rate*, number of deceased per 1000 people | 13.8 | 2.0 | 10.5 | 17.0 |
| **Social Characteristics** | | | | |
| Gini Index* | 0.39 | 0.02 | 0.36 | 0.44 |
| Unemployment rate according to the Federal Labour and Employment Service*, % | 1.63 | 0.57 | 0.62 | 2.96 |
| Number of private passenger cars at the year-end*, per 1000 people | 258 | 36 | 204 | 336 |
| Total floor area per 1 citizen at the year-end*, square meters | 23.9 | 2.0 | 21.4 | 27.0 |
| Decrepit and dilapidated housing in the total floor area of all the available housing*, % | 3.05 | 1.31 | 0.98 | 5.20 |
| Proportion of students of state and municipal general education institutions (excluding evening schools), second and third shifts, at the beginning of the academic year*, % of the total number of students | 14.7 | 5.3 | 9.3 | 23.6 |
| Number of Bachelor’s, Specialist’s, and Master’s degree students*, per 10,000 people | 400 | 118 | 207 | 683 |
| Number of theater-goers*, per 1000 people | 215 | 53 | 147 | 305 |
| Newspapers printed per 1000 people*, print run, copies | 867 | 238 | 372 | 1285 |
| Number of recorded crimes*, per 100,000 people | 1854 | 513 | 902 | 2525 |
| Density of public hard-surfaced roads at the year-end*, kilometers per 1000 km^2^ of the territory | 175 | 162 | 10 | 596 |
| Number of traffic accidents*, per 100,000 people | 153 | 43 | 86 | 229 |
| Number of research and development organizations*, per 100,000 people | 2.13 | 1.01 | 0.98 | 4.76 |
| **Economy** | | | | |
| Per capita income per month*, in rubles | 19 106 | 2 817 | 15 735 | 23 920 |
| Population with income below the minimum subsistence level*, % | 14.0 | 2.0 | 11.1 | 16.8 |
| Fixed asset value at full cost at the year-end*, million rubles | 1 329 499 | 623 407 | 188 546 | 2 163 972 |
| Depreciation rate of fixed assets at the year-end*, % | 45.1 | 7.5 | 28.1 | 57.4 |
| Per capita retail turnover*, at effective prices, in rubles | 122 696 | 26 919 | 98 422 | 181 820 |
| Per capita amount of paid services*, in rubles | 36 326 | 8 139 | 27 882 | 57 789 |
| Per capita capital investments*, at effective prices, in rubles | 80 958 | 38 308 | 29 448 | 170 937 |
| Per capita Gross Regional Product**, in rubles | 265 784 | 91 893 | 126 366 | 416 468 |
| Per capita actual final consumption of households **, in current market prices, in rubles | 181 712 | 26 128 | 147 439 | 223 548 |
| **Environmental and Industrial Characteristics** | | | | |
| Emissions of pollutants from stationary sources into the atmosphere*, 1000 tons | 512.9 | 724.6 | 4.4 | 2 488.8 |
| Volume of shipped own-produced goods, in-house works, and services related to mineral extraction*, million rubles | 123 085 | 150 572 | 407 | 451 893 |
| Volume of shipped own-produced goods, in-house works, and services related to manufacturing*, million rubles | 336 423 | 239 376 | 15 658 | 721 966 |
| Volume of shipped own-produced goods, in-house works, and services related to electric power, gas, and water production and distribution*, million rubles | 55 886 | 32 474 | 6633 | 108 173 |
| Agricultural products of farms of all categories*, million rubles | 48 789 | 32 312 | 13 297 | 119 602 |
| Average annual number of employees of fisheries and aquaculture sectors*, number of people | 1271.0 | 3509.4 | 14.8 | 12 396.6 |
| Timberland area*, % | 38.1 | 26.4 | 4.3 | 77.3 |
| Proportion of people employed in toxic and/or hazardous jobs—in the mineral extraction, manufacturing, electric power, gas, and water production and distribution, construction, transport and communication sectors—of the total workforce, at the year-end of 2014, for all forms of incorporations, % | 43.0 | 12.0 | 22.6 | 66.8 |
| **Alcohol Consumption** | | | | |
| Sales of vodka, liqueurs, and spirits*, liters per capita | 9.0 | 3.2 | 2.6 | 13.4 |
| Sales of brandy and brandy spirits*, liters per capita | 0.64 | 0.23 | 0.24 | 1.12 |
| Sales of low-alcohol beverages, with alcohol content of under 9%, excluding wines, champagne, and beer*, liters per capita | 1.94 | 1.46 | 0.12 | 6.00 |
| Sales of wine-making products, excluding champagne and sparkling wines*, liters per capita | 6.22 | 2.15 | 1.34 | 9.50 |
| Sales of champagne and sparkling wines*, liters per capita | 1.59 | 0.50 | 0.54 | 2.48 |
| Sales of beer*, liters per capita | 67.67 | 19.55 | 18.70 | 87.82 |
| **Medical Infrastructure Characteristics and Health Status** | | | | |
| Population size per 1 hospital bed at the year-end*, people | 106.4 | 11.4 | 89.7 | 134.0 |
| Population size per 1 doctor at the year-end*, people | 196.7 | 35.3 | 139.6 | 286.5 |
| Abortions per 100 women of 15–49 years old*, number | 35.3 | 9.5 | 20.6 | 50.6 |
| Diseases registered in patients with a diagnosis made for the first time in their life*, per 1000 people | 781 | 129 | 540 | 1 004 |
| Number of visits to outpatient clinics at the year-end, per 1 shift per 10,000 people * | 265.1 | 27.9 | 235.7 | 316.6 |
| Life expectancy*, years | 69.4 | 1.7 | 66.8 | 73.3 |
| Mortality rate from infections and infestations*, per 100,000 people | 26.8 | 13.5 | 10.3 | 49.7 |
| Mortality rate from tuberculosis*, per 100,000 people | 14.3 | 7.7 | 6.2 | 29.0 |
| Mortality rate from oncology-related diseases *, per 100,000 people | 205.6 | 29.8 | 129.0 | 224.8 |
| Mortality rate from circulatory diseases*, per 100,000 people | 718 | 120 | 495 | 903 |
| Mortality rate from diseases of the respiratory system*, per 100,000 people | 59.0 | 17.0 | 22.7 | 93.5 |
| Mortality rate from diseases of the digestive system*, per 100,000 people | 74.9 | 17.4 | 48.5 | 118.5 |
| Mortality rate from external causes*, per 100,000 people | 150.1 | 35.8 | 68.1 | 214.0 |

* average for 2010–2014; ** average for 2010–2013.
